# T Cell Epitope Prediction and Its Application to Immunotherapy

**DOI:** 10.3389/fimmu.2021.712488

**Published:** 2021-09-15

**Authors:** Anna-Lisa Schaap-Johansen, Milena Vujović, Annie Borch, Sine Reker Hadrup, Paolo Marcatili

**Affiliations:** Department of Health Technology, Technical University of Denmark, Lyngby, Denmark

**Keywords:** epitope prediction, neoantigens, neoepitope prediction, T cell, TCR, T cell receptor

## Abstract

T cells play a crucial role in controlling and driving the immune response with their ability to discriminate peptides derived from healthy as well as pathogenic proteins. In this review, we focus on the currently available computational tools for epitope prediction, with a particular focus on tools aimed at identifying neoepitopes, i.e. cancer-specific peptides and their potential for use in immunotherapy for cancer treatment. This review will cover how these tools work, what kind of data they use, as well as pros and cons in their respective applications.

## Introduction

T cells recognize and survey peptides (epitopes) presented by major histocompatibility complex (MHC) molecules on the surface of nucleated cells. To be able to perform this task, T cells must be able to differentiate between native “self” peptides versus peptides deriving from pathogens, infections or genomic mutations. In order to effectively mount and initiate an immune response, T cells must undergo activation. The main requirement of T cell activation is the molecular recognition between the T cell receptor (TCR) expressed on the T cell surface and peptide-MHC complexes (pMHC) presented on the surface of other cells. This precise recognition process is of paramount importance for a well-functioning immune system, and is shaped by a mechanism named central tolerance. In order to ensure that T cells do not react against ubiquitous peptides found in an individual, T cells undergo the process of negative selection. Early in their development, T cells are presented with a plethora of self-peptides, where any T cell that recognizes self-peptides is eliminated, leaving only T cells with little or no specificity for self. Cases in which this mechanism fails and T cells recognize self-epitopes are typically associated with harmful effects on the organism and might result in autoimmune disorders.

As mentioned earlier, T cells recognize epitopes only when they are presented by MHC molecules. Early in the thymic development of T cells, they undergo the process of positive selection ensuring that they bind to host MHC molecules. There exist two classes of MHC molecules: class I expressed on surfaces of all nucleated cells and class II found on surfaces of specialized antigen-presenting cells (APCs). As two classes of MHC molecules occur, two types of T cells are specially equipped for binding to the MHC I and II, the CD8+ and CD4+ T cells, respectively. The general focus of this review will be on cytotoxic CD8+ T cell binding to MHC I presented epitopes.

**Figure 1:**
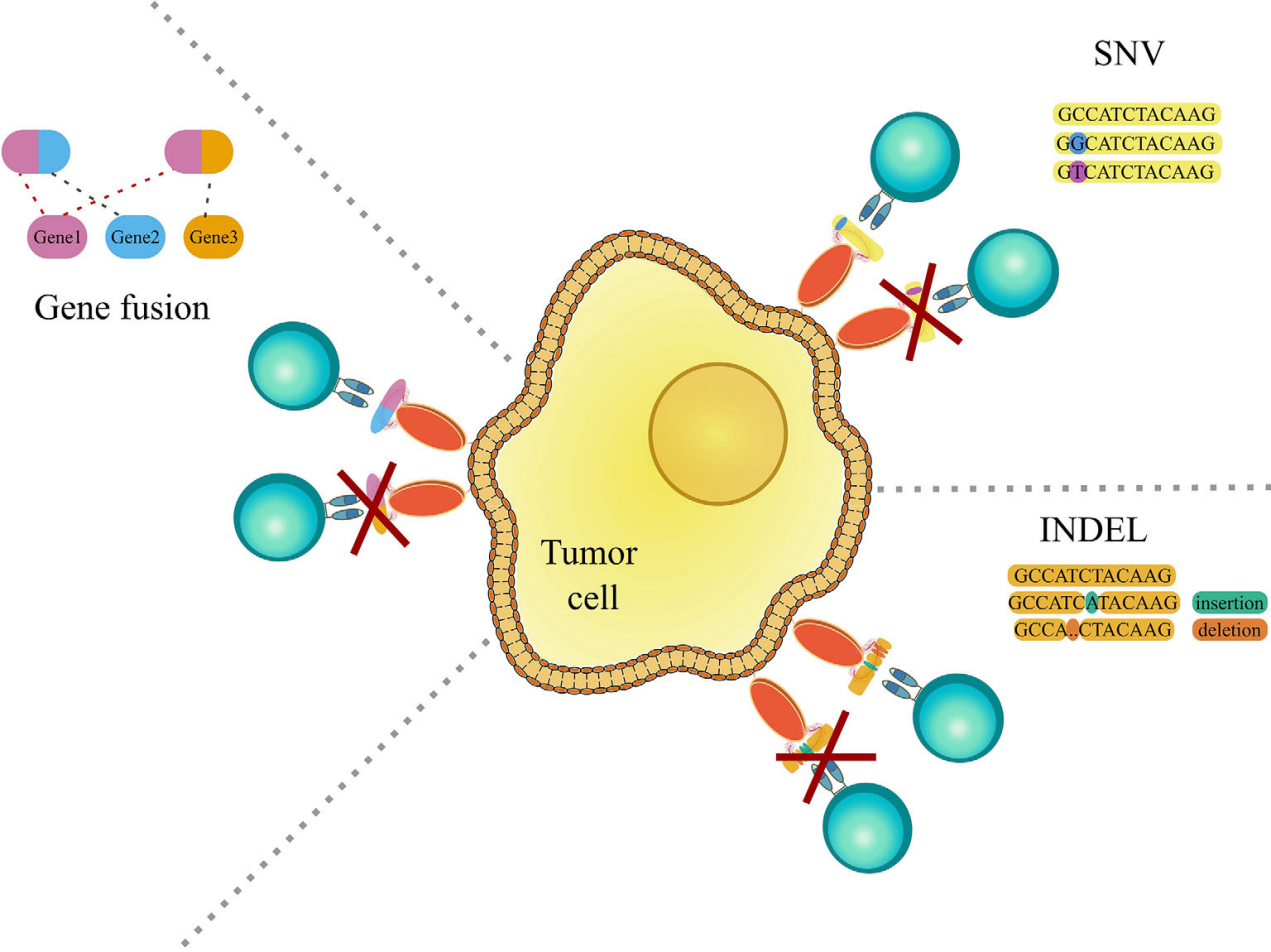
Graphic representation showing genomic aberrations, which can lead to the occurrence of cancer-specific peptides (neoepitopes). The left panel shows gene fusions, which is the rearrangement of two genes leading to the encoding and translation of a potentially novel immunogenic peptide. The right upper panel shows single nucleotide variations (SNV) and the right lower panel shows insertions and deletions (indels), that may cause the creation of immunogenic cancer-specific peptides. For further detail see the main text.

The immune system in general is very good at identifying “foreign” peptides stemming from bacterial or viral infections. On the other hand, as initially proposed by Burnet and Thomas through the idea of immunosurveillance (1, 2), the same process can also protect our organism from cancer, by recognizing cancer-specific peptides (neoepitopes) generated by somatic mutations or genomic aberrations (**Figure 1**). The ability of the immune system to target cancer cells has been exploited by a novel class of therapies, such as adoptive T cell therapy and cancer vaccines, named immunotherapies. These approaches, by exploiting the high selectivity of the immune system, have the advantage to be more specific and less invasive than traditional cancer therapies, and potentially effective even at later stages by providing immunological memory.

Broadly, immunotherapy can be divided into two categories: “active” and “passive”. The “active” works to stimulate T cells of the individual’s immune system into attacking tumor cells i.e. effectively training the immune system *in vivo*. The “passive”, focuses on *in-vitro* training and subsequent injection of immune agents that will help battle the disease *in vivo* (3). Passive immunotherapy includes therapies such as adoptive cell therapy, cytokine injection, monoclonal antibodies and lymphocytes (4, 5). Active immunotherapies encompass therapies such as non-specific immunomodulation and vaccination (6, 7).

Computational tools for epitope prediction have been recognized as being crucial for successful development of various cancer immunotherapies (8). This review will therefore give an overview of both general and cancer specific epitope prediction tools and discuss the pros and cons of the different tools and future perspectives in the field.

## Epitope Prediction Methods

**Figure 2:**
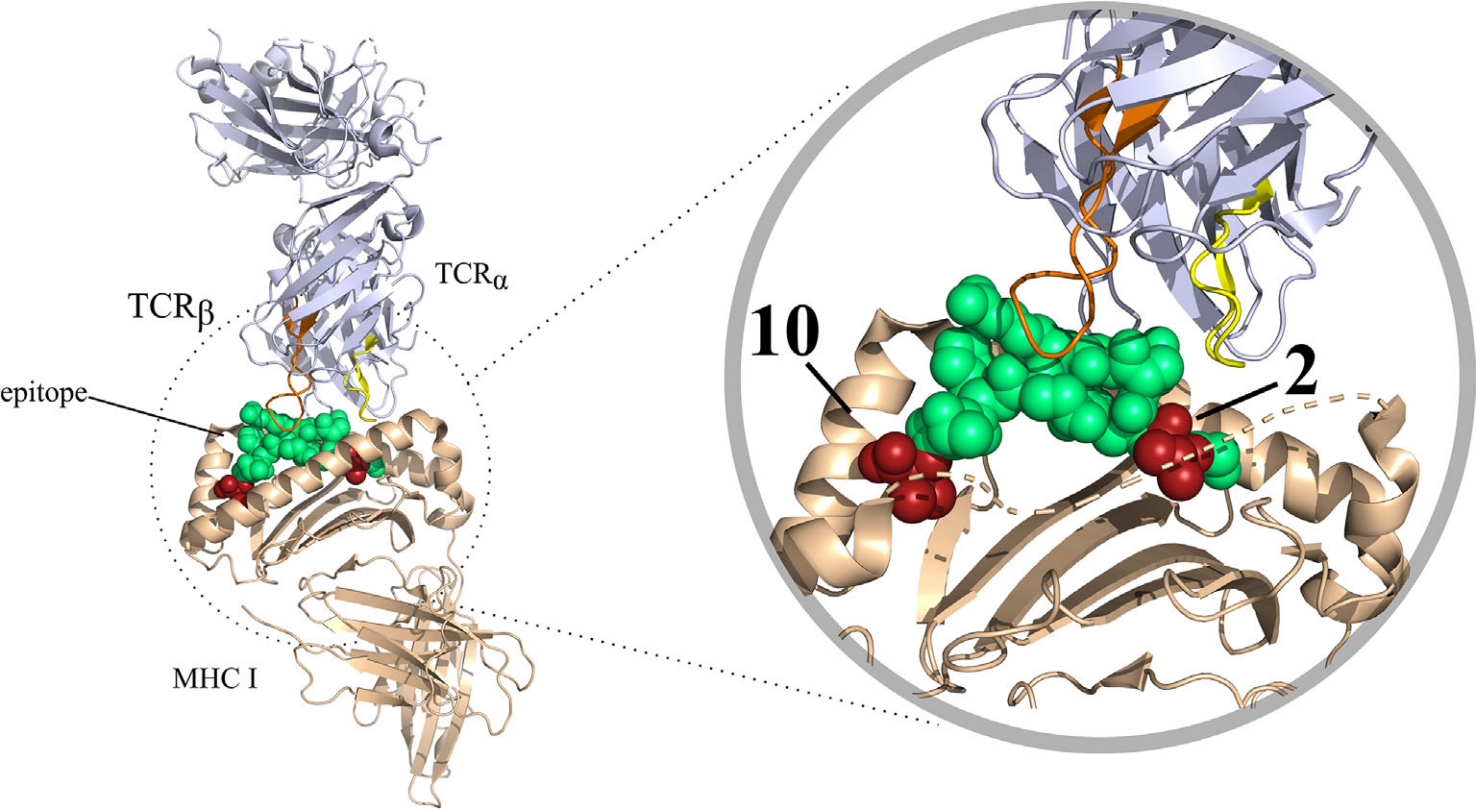
T-cell interaction with a pMHC complex rendered in PyMOL (PDB code: 6TRO). Here MHC I is shown as colored in beige. The TCR is colored blue white. The CDR3 variable regions of the T-cell have been colored in different colors, these are as follows: CDR3 α colored in yellow, CDR3β colored in orange. The bound peptide is colored in green, with the anchor residues are colored in red.

As mentioned before, a peptide needs to be presented by an MHC I molecule for it to be able to elicit effector T cell responses. Contrarily to MHC II molecules, which can bind to peptides that are longer and more variable, MHC I binding is restricted to peptides typically 8-14 amino acid long in sequence and that some of the residues in the peptide, denoted anchor residues, are important for peptide-MHC binding (9) (**Figure 2**). In most human alleles the anchors are the second and the last residues in the peptide (10), but this depends on the allele and species. The binding of peptides to MHC molecules is therefore a very selective step, which has been a major focus in many epitope prediction models. However, most peptides presented by MHC molecules will not elicit an immune response as they do not evoke TCR specific recognition by the T cell. In order to shed light on this interaction, computational models are being constructed with the goal of predicting T cell recognition of the presented peptide and its connection to an overall immune response. Epitope prediction can thus currently be divided into two main focus areas. The first addresses the presentation of peptides by MHC molecules. Extensive reviews on this subject have been published recently, and we single out the in depth work by Peters et al. (11). In this review, we mainly focus on the second part of the interaction: predicting T cell recognition of pMHC complexes.

One of the first attempts at defining the immunogenic potential of peptides was based on their local and global physico-chemical characteristics, regardless to the specific T cell interaction. One of such tools is POPI (12), which is a support vector machine (SVM) based method. SVMs are machine learning tools that can identify complex non-linear relationships between the input data and the predicted variable. In this case, a feature set of physico-chemical properties derived from MHC I binding peptides is used to predict the peptide’s immunogenicity. POPI uses averaged values of the physico-chemical properties independent of the amino acid positions in the peptides, therefore being unable to take local information into consideration in the predictions.

Another model named POPISK (13), by the same group, tries to improve on this by utilizing a SVM in conjunction with a weighted degree string kernel. The model is seemingly only capable of predicting immunogenicity for HLA-A2-binding peptides. Where predictions reached an overall accuracy (ACC) of 0.68 and 0.74 for area under the curve (AUC). The ACC and AUC are calculations based on a confusion matrix, which in different ways essentially estimates how often an algorithm predicts correctly. In both cases, a perfect prediction would have both ACC and AUC equal to 1, and lower values for worse predictions. A more exhaustive introduction to accuracy metrics for prediction tools can be found in Peters et al. (11). It should be mentioned that the dataset was not pre-processed to remove or reduce the redundancy - i.e. very similar peptides might be present. This has been observed to have a negative impact on the methods’ ability to generalize, that is the ability of an algorithm to achieve good results on data that is different from the data used to train. A typical strategy to deal with this issue is to perform some form of homology reduction to reduce redundancy. In the discussion we will discuss more about the importance of such procedure when assessing the actual accuracy of prediction tools. Furthermore, it should be noted that both POPI and POPISK are not available for general use anymore.

Calis et al. created the immunogenicity model (14) based on experimental indications. The authors discovered that T cells show a preference for binding peptides containing aromatic and large amino acids. They also showed that positions 4-6 were important in regards to immunogenicity. Based on this information, a scoring model was created which scores peptides based on the ratio of an amino acid between a non-immunogenic and immunogenic dataset. Furthermore, it weights the amino acid based on its position in the ligand. The authors estimated the accuracy of the model on new MHC I binding peptides, and obtained an AUC of about 0.65, thus the model is only to some extent predictable. It should be noted, that where models such as POPISK only is capable of predicting TCR propensity for HLA-A ^*^02:01, the Calis et al. immunogenicity model can make predictions for any MHC I molecule.

PAAQD (15) is a model which focuses on predicting T cell reactivity. It works by encoding nine-mer peptides which are processed in a random forest algorithm, in order to predict the immunogenicity of a peptide binding to MHC I. The peptides are numerically encoded by combining information regarding quantum topological molecular similarity (QTMS) descriptors and amino acid pairwise contact potentials (AAPPs). In the article it was mentioned that an ACC of 0.72 and a AUC of 0.75 was obtained for immunogenicity prediction. It obtained a higher AUC and ACC than POPISK and a higher AUC than the immunogenicity model by Calis et al., however, like POPISK, no homology reduction was done to reduce redundancy. Furthermore the model had a focus on HLA-A2 and will have limited success in predicting immunogenic peptides for other HLA molecules.

Jørgensen and Ramussen, who developed NetMHCstab (16) and NetMHCstabpan (17) respectively, theorized that instead of entirely focusing on the HLA binding affinity one should also take pMHC stability into account to predict immunogenic MHC I ligands. They based this hypothesis on the assumption that a more stable presentation of an epitope bound to an MHC will increase the likelihood of a T cell recognizing the epitope. However, as the authors have also indicated in the papers themselves, stability alone did not give as good results as combining a stability predictor with a pMHC I binding predictor.

Experimental investigation of peptide presentation and binding by Schmidt et al. (18) showed poor correlation with predictions for the same peptides by NEtMHCstab and NetMMHCpan in combination with a binding affinity predictor. These models were outperformed by another epitope prediction model: NetTepi (19). This model has been built on top of previous efforts and combines: peptide-MHC stability using NetMHCstab, T cell propensity predictions using the immunogenicity model by Calis et al. and peptide-MHC binding affinity using NetMHCcons (20). The model has been stated to be capable of predicting T cell epitope for multiple HLA molecules with a sensitivity of 90% and a false positive rate of 1.5%.

One of the newer models for predicting which epitopes will be recognized by T cells is NetTCR (21). NetTCR implements a convolutional neural network (CNN) model to predict TCR recognition of a peptide. CNNs are a type of neural network which are very popular for different tasks (e.g. image recognition) and capable of identifying local patterns in the input data. The model takes as input a HLA-A ^*^02:01 binding MHC I peptides and the CDR3 protein sequence of a T cell receptor. The model obtained a somewhat high AUC of 0.727. The AUC is lower than the AUC for POPISK (0.74) and PAAQD (0.75). However, it should be noted that unlike POPISK and PAAQD, NetTCR performed homology reduction to reduce any redundancy in the data.

A major bottleneck in improving the accuracy of models is in the limited amount of available training data. However, several databases collecting experimental immunogenicity data are now available, with one of the first to pioneer this area being SYFPEITHI from Rammensee et al. in 1999 (22). Newer databases have since been created such as IEDB (23), VDJdb (24), McPAS-TCR (25), ATLAS (26) and STCRDab (27). The steadily increasing amount of experimental data will support the generation of models with greater prediction power.

## Structural Epitope Prediction

The energetic balance of the TCR-pMHC interaction is one of the main drivers in dictating the initiation of an immune response. As evident from structural (28) and mutagenesis studies (29), this balance is very delicate. All circulating T cells have undergone the so-called positive selection process, meaning that they must bind with low affinity to MHC molecules, regardless of the specific epitope. Additionally, TCR interaction is highly cross-reactive, meaning that a single TCR will potentially be able to bind to thousands of peptides. This poses a serious hurdle to develop computational tools to predict immunogenicity based on structural calculations. In recent years, it has been shown that, when using fine-grained molecular dynamics (MD) simulations, one can to some extent predict TCR-pMHC interactions (30). Unfortunately, this approach is neither very precise nor feasible. For such calculations, high quality structures of the interacting molecules are needed, and the current available amount of solved structures for TCRs is very limited - less than three hundred at the time of writing. In contrast, the number of different TCRs that circulate at any time in humans is 10^6^ to 10^8^ (31), and the theoretical numbers of different TCRs is at least 4 x 10^11^ (32). This stark difference greatly reduced the usefulness of such methods to a tiny minority of the available cases. Even when solved structures are available, MD simulations are very demanding in terms of computing time. The dynamics of the TCR-pMHC interaction, especially regarding their dissociation rate, have time scales that are currently at the very limit of what one can achieve with full-grain MD Simulations.

Some works have focused on solving these 2 problems - the lack of structural information and the need for more efficient structure-based algorithms. It is now possible to model to a very good accuracy TCRs, pMHCs, and their complexes. Without delving in too much detail, most currently available methods (33–35) can model pMHC complexes to a very good accuracy - often less than 1Å Root Mean Square Deviation (RMSD) - from the native structure, and almost as good as the experimentally resolved structures. TCRs can also be modeled with good accuracy (in general less than 2Å RMSD), with some minor exception for the CDR3 regions of both TCR chains. The real culprit of all modeling tools is in predicting the correct mutual orientation of the TCR with respect to the pMHC, for which only a decent accuracy can be achieved: approximately, only 50% of the molecular contacts between TCRs and pMHC are recovered in the model. Given the current accuracy of the modeling tools for TCR-pMHC complexes, together with the computational cost of running detailed atomistic simulation, underline the need of more coarse-grained models, that can ease both the aforementioned problems. In recent years, Lanzarotti and co-workers (36, 37) used TCR-pMHC models to refine existing computational force fields [Rosetta (38) and FoldX (39)], and combined such refined energy calculations in a simple statistical framework to improve the prediction of existing TCR-pMHC complexes. The authors show that, even in such a simple approach, it is possible to exploit structural models to identify, among a pool of TCRs and pMHCs, the actual interacting partners. The same results have recently been confirmed using a similar approach (40). The authors show that, by investigating the energy and the structural variability in TCR-pMHC models, it is possible to improve the prediction of TCR-pMHC pairs. At the current stage, structure-based methods can greatly reduce the number of false positive predictions obtained by sequence-only methods, at the cost of reduced sensitivity.

## Neoantigen Prediction

Genome aberrations are a typical feature of many cancer types (41). On the one hand such aberrations are linked to the cancer occurrence and growth, i.e. by disrupting normal cell cycle and apoptosis control. On the other hand, they can be exploited by the immune system to recognize and eliminate cancer cells. As mentioned previously, neoepitopes have been a major target of immunotherapy approaches such as adoptive T cell therapy or cancer vaccination. Several computational tools have been developed to assist and improve immunotherapy. The main rationale of these tools is to first identify aberrations in the cancer genome, and then, to a different extent and with individual approaches, to predict the ones that are more likely to trigger an effective immune response. Besides genomic aberrations, events such as post-translational modifications (PTMs) (42) and peptides derived from non-coding regions (43) can also cause neoepitopes to arise. However, due to the limited availability of data and of the biological basis of these, there are currently only very few computational tools for their analysis and prediction (44). Broadly speaking, the available tools can be categorized by the type of input data they accept, by the type of variants they can call, and by the strategy used to filter or prioritize the most immunogenic variants. Regarding the first point, neoepitopes can arise due to events such as single nucleotide variations (SNV), insertions and deletions (indels), intron retention, and chromosomal aberrations (45–48). While most of the tools can predict neoepitopes from SNVs [Epi-Seq, TIminer, Neopepsee, DeepAntigen], some also incorporate indels [pVACseq, MuPeXI, Epidisco, OpenVax, NeoEpiScope, CloudNeo, pTuneos, antigen.garnish, NeoPredPipe, TSNAD], and others only focus on indels [ScanNeo], gene fusions [NeoFuse, INTEGRATE-neo], or they let the users input the variants as peptides [EDGE, DeepHLApan], for an overview see **Table 1**. Another difference between the tools is the types of data that these models rely on. In most cases the tools use whole genome sequencing (WGS), whole exome sequencing (WES), transcriptome sequencing (RNA-seq), peptide sequencing, or a combination of those. Finally, in order to filter and prioritize neoepitopes, many tools incorporate predictions from NetMHC (68) and NetMHCpan (69), alongside some other tools for predicting MHC binding. In the following, we will briefly present the available tools based on the characteristic that we have just discussed.

**Table 1:** Overview of the different neoantigen prediction tools.

Bioinformatic tools for neoantigen prediction							
Tool	DNA	RNA	Peptide	SNV	indels	Gene fusion	Reference
Epi-seq		X		X			(49)
TIminer	X	X		X			(50)
Neopepsee	X	X		X			(51)
DeepAntigen	X	X		X			(52)
PVACseq	X	X		X	X		(53)
Mupexi	X	X		X	X		(54)
Epidisco	X	X		X	X		(55)
OpenVax	X	X		X	X		(56)
Neoepiscope	X	X		X	X		(57)
CloudNeo	X	X		X	X		(58)
pTuneous	X	X		X	X		(59)
antigen.garnish	X	X		X	X		(60)
NeoPredPipee	X	X		X	X		(61)
TSNAD	X	X		X	X		(62)
ScanNeo		X			X		(63)
NeoFuse		X				X	(64)
INTEGRATE-neo	X	X				X	(65)
EDGE		X	X	X	X	X	(66)
DeepHLApan			X	X	X	X	(67)

### Single Data–Based Models

Both RNA-seq and DNA-seq data can be exploited to identify variants in the cancer genome, and several tools make use of these data to predict neoantigens. It is important to notice that these two experimental methods provide complementary information. DNA-seq data is in general more sensitive, i.e. it can identify more variants. RNA-seq experiments can be used to generate expression levels at the gene or, as at the transcript level, thus helping to prioritise variants that are present in highly abundant genes over those that have low or no expression. It should be noted that the transcript level is often recommended, since this can further give information regarding events important for neoepitope prediction, such as isoform selection and alternative splicing (70–72). Peptide sequencing can also be used for neoantigen prediction. This holds information regarding whether a gene is actually expressed or not at the protein level. This is very important information; identified variants at DNA or RNA level are not always expressed at protein level. The reader should take this into account when deciding which tools they want to use.

Epi-Seq (49) is a tool which only uses tumor RNA-seq data. Epi-Seq works as a wrapper tool, i.e. it combines the output of other tools to perform an integrated prediction. It only supports SNV variant calling and neoantigen prediction from those calls. The Epi-Seq pipeline is very useful when only RNA-seq data is available. However, since the pipeline only focuses on SNV variants other potentially important variants are not predicted on.

ScanNeo (63) is a tool capable of predicting neoepitopes from small to large-sized indels. ScanNeo is a wrapper tool, which takes as input RNA-seq data. The three major steps in its pipeline are i) indels discovery, ii) annotation and filtering and iii) neoantigen prediction. ScanNeo uses NetMHC in its pipeline. Besides NetMHC, the tool also employs NetMHCpan in its pipeline to predict peptides that bind to HLA class I with high affinity.

NeoFuse (64) is a computational pipeline predicting neoantigens from gene fusions. It is a wrapper tool which uses raw RNA-seq data from patient tumors as input to do HLA class 1 typing, predict fusion peptides and quantification of gene expression. MHCflurry (73) to predict pMHC binding and the gene expression levels are utilized to filter out candidate fusion neoantigens. Like Epi-seq this is convenient when only tumor RNA-seq data is available.

DeepHLAPan (67) is a recurrent neural network-based approach, which takes both peptide-HLA binding and potential peptide-HLA immunogenicity into account. The tool predicts neoepitopes utilizing HLA class I typing provided by the user and peptides. The tool further filters the candidate neoantigens based on a score generated by an immunogenicity model based on immunogenicity data from IEDB.

### Data Integration–Based Models

Next generation sequencing (NGS) has made it easier to sequence in parallel the DNA and RNA of a patient. By integrating the use of both DNA and RNA data, the researcher can call somatic mutations from the DNA and quantify gene and transcript expression from the RNA data, which can help in identifying which variants are more likely to be expressed. Also in this case, most of the computational tools are in fact wrappers of multiple different methods which are integrated in multi-step workflows to perform the neoepitope prediction. Besides integrating DNA and RNA data, it is also possible to predict neoepitopes from peptide and RNA sequencing data. The peptide data enables us to know which genes are actually expressed at protein level and the RNA data helps with identifying which of the peptides will be presented by the HLA alleles, since expression of messenger RNA is strongly correlated with HLA peptide presentation (74). In general integrating data can often help in generating more accurate predictions, as many of the tools which will be mentioned in this section also have shown in their studies. When choosing tools, the reader should keep in mind the somatic variations they want to account for and what kind of data they possess.

pVACseq (53) is a neoantigen prediction tool, which can work with either WES or WGS data together with RNA data. This tool can predict neoantigens from small indels and SNVs. pVACseq utilizes HLAminer (75) to infer the patients HLA class I typing and NetMHC to predict HLA class I restricted epitopes. The tool prioritizes neoepitopes based on sequencing depth and fraction of reads containing the variant allele.

INTEGRATE-neo (65) is another tool which also uses NetMHC in its pipeline. This tool is based on INTEGRATE (76), which uses DNA sequencing data to predict peptides generated by gene fusion events, and thereafter uses HLAminer to perform in silico HLA typing, and lastly uses NetMHC to predict neoantigens based on the gene fusions. Where the other tools can work just with the DNA data, optionally also integrating RNA data into their pipelines, INTEGRATE-neo requires the use of both DNA and RNA. A tool suite named pVACtools which includes pVACseq and INTEGRATE-Neo among other tools to not only account for SNVs and small indels but also include support for structural variants.

MuPeXI (54) like pVACseq requires the user to provide HLA types, somatic variants and optionally gene expression estimates. The tool predicts neoantigens from SNVs and indels. The tool can use either WES or WGS data and optionally also RNA data and have similar features to pVACseq. However, unlike pVACseq, MuPeXI also offers i. a priority score to rank peptides ii. a comprehensive search for self-similarity peptides and lastly iii. besides being a downloadable command-line tool it is also available as a webserver. Furthermore, this model incorporates the use of NetMHCpan (69) in its pipeline instead of NetMHC.

Epidisco (55) takes as input wild type DNA, tumor DNA and tumor RNA sequencing data. The tool maps the normal and tumor DNA samples to the human GRCh37 reference genome. Epidisco, like many of the other tools mentioned works as a wrapper around other existing tools, and also like many of the other tools, Epidisco uses NetMHCpan in its pipeline. The tool supports SNV and indel based neoantigen prediction. Epidisco focuses on vaccine peptide selection, and generates a ranked list of peptide candidates.

TIminer (50), like many of the other tools, is a tool which as input requires a pre-existing set of variants derived from DNA. The tool also incorporates NetMHCpan in its pipeline and unlike other tools it is able to process raw RNA-seq data which may obtain more information relevant for neoantigen prediction. This tool, however, only supports neoantigen prediction from SNVs.

OpenVax (56) is another pipeline which integrates the use of NetMHCpan into its pipeline, however, it is also possible to choose other MHC binding peptide predictors. The OpenVax pipeline, unlike many of the other tools takes as input raw DNA and RNA sequencing files. The OpenVax pipeline has also included somatic variant calling tools in its pipeline which are capable of calling SNVs and indel variants. It has a ranking function similar to MuPeXI, but with less features, namely MHC class I affinity scores and RNA-seq read count based variant expression.

NeoEpiScope (57) is another tool which can use NetMHCpan in its pipeline. The tool in general uses MHCflurry or MHCnuggets, however, NetMHCpan can also be used if installed individually. Like many of the other tools, NeoEpiScope requires as input a set of somatic variants and supports SNV and indel based neoantigen prediction. The main focus of this tools is to prioritize handling phased variants. To use the phasing function, the user must submit patient haplotypes.

CloudNeo (58) is a tool developed for cloud computing, created to eliminate the need for local infrastructure investment in computation, data storage and transfer, while also providing scalable computational capabilities for neoantigen identification. CloudNeo is a wrapper like many of the other tools which also utilizes NetMHCpan in its pipeline. CloudNeo supports SNVs and indels for neoantigen prediction. Although CloudNeo uses RNA data in its pipeline, it seemingly only utilizes the RNA data for HLA typing, however, DNA data can also be used for this purpose.

Neopepsee (51) is a tool which takes as input a list of somatic mutations and raw RNA seq data. The tool focuses on non-synonymous somatic mutations and works as a wrapper tool, which uses tools such as NetMHCpan to predict MHC binding affinity. For peptides with the highest binding affinity, immunogenicity features are then calculated and fed into a locally weighted naïve Bayes classifier. The idea with Neopepsee is to use a classifier to decrease the amount of false-positives that using only binding affinity would provide.

pTuneos (59) predicts and prioritizes candidate neoantigens from SNVs and indels. The tool is a wrapper tool, which takes as input raw WGS/WES tumor normal matched sequencing data and optionally also tumor RNA-seq. The tool utilizes HLA class I typing and NetMHCpan to predict binding affinity of normal and mutant peptides, which is then run through a random forest model to predict a T cell recognition probability. Finally they use a scoring schema to evaluate whether a candidate neoepitope that can be recognized by a T cell will be naturally processed and presented. This can be used to prioritize the peptides based on *in vivo* immunogenicity.

The package antigen.garnish (60) is an wrapper tool in R, utilizing NetMHCpan among others for peptide MHC binding in its pipeline. It predicts neoantigens from SNVs and indels. Besides MHC binding it also takes hydrophobicity, comparison of MHC binding affinity between mutated and non-mutated counterpart, and dissimilarity into account. Furthermore, the tool also calculates a TCR recognition probability based on the dissimilarity.

NeoPredPipe (61) is another tool which incorporates NetMHCpan into its pipeline. Like many of the other tools the user has to submit files regarding patient haplotypes and SNVs and indels. NeoPredPipe unlike the other tools provides the opportunity of neoantigen prediction on multi-region sequencing data and also asses the intra-tumor heterogeneity, which is done based on multi-region samples, where the neoantigen burden is reported for clonal, subclonal and shared variants. NeoPredPipe furthermore also predicts the likelihood of TCR recognition. This based on the probability of the mutant epitopes ability to bind to MHC I molecules and the epitopes similarity to pathogenic peptides.

TSNAD (62) is a tool which earlier had netmhcpan integrated in its pipeline, however, in their version 2.0, which was updated in 2019, they replaced NetMHCpan with the earlier mentioned DeepHLAPan to predict binding of the mutant epitopes to MHC I molecules. TSNAD works by, like many of the other tools by integrating multiple tools into its pipeline. The tool takes as input raw read of tumor normal DNA pairs. The sequences can either be mapped to GRCh37 or GRCh38. In the updated version, raw RNA-seq data can optionally be added to help filter neoantigens. The tool supports neoantigen prediction from SNVs and indels.

DeepAntigen (52) is a deep sparse neural network model based on group feature selection (DNN-GFS). Uniquely this model bases its predictions on the DNA loci of the neoantigens in a 3D genome perspective. The authors discovered that the DNA loci of the immunonegative and immunopositive MHC class I neoantigens have distinct spatial distributions. The model uses preprocessed WES and messenger RNA-seq for calling somatic mutations and estimating gene expression. The model also takes as input Hi-C (77) data (captures chromosome conformation) for 3D genome information. However, this method can only predict neoepitopes from non-synonymous point mutations and 9 mer peptides.

EDGE (66) is a commercial platform for neoantigen identification. The EDGE model is a neural network trained on HLA peptide mass spectrometry data and RNA-seq data from various human tumors. The model uses HLA class I type and sequence, RNA and peptide sequencing data or peptides generated from somatic variant calling data to predict neoantigens. Although the model does not incorporate TCR binding, it is still to a certain extent able to capture T cell recognition with the addition of RNA expression.

## Discussion

In recent years, the number of computational tools for epitope and neoepitope prediction has exploded. In many cases, these tools combine the results of other methods, using different heuristic approaches, to perform their predictions. Unfortunately, the amount and quality of available data make it difficult to decide which of these approaches are sound, and which are not. As an example, many of the currently existing epitope and neoepitope prediction methods are mainly focusing on MHC presentation. This is because, from a quantitative point of view, MHC binding is the most selective step. According to Yewdell et al. around 1 in 200 peptides bind to MHC class I with an affinity strong enough (500 nM or lower) to induce a immune response (78). Other studies, such as Sette et al. (79), also indicated an MHC affinity threshold of 500 nM to be associated with T cell recognition of HLA class I bound peptides. Moreover, MHC binding is considered necessary but not sufficient for a molecule to be immunogenic: in general only the minority of epitopes predicted are immunogenic (80–82). However, this paradigm has been challenged on many occasions. In particular for neoepitopes, there is not a general consensus on the fact that a strong MHC binding is connected to immunogenicity. A recent study by Bjerregaard et al. (83), supports the theory that strong binders are immunogenic. Their study indicated that immunogenic neopeptides bind significantly stronger compared to non-immunogenic peptides and that they in general bind with a strong affinity. However, Duan et al. (49) deemed binding affinity scores alone, especially from NetMHC, as not being an effective predictor of tumor rejection and immunogenicity. In fact, in their study they noticed that the epitopes that did elicit tumor protection were in general not strong MHC class I binders. They therefore created an algorithm which subtracts the predicted NetMHC scores of unmutated counterpart peptides from the NetMHC scores of the mutated peptides. This setup is referred to as the differential agretopicity index (DAI). The idea is that this can reflect to which degree the binding of mutated peptides differ from their unmutated counterparts (49). Even this score, however, performed poorly for identifying effective neoepitopes (84). Similar indications have also been made by (85) and (86), where it was shown that not only peptides predicted as strong binders but also peptides predicted as weak binders or non-binders are capable of initiating a T cell response. At the current stage, there’s no clear consensus on the importance of MHC binding for identifying dominant epitopes and neoepitopes. Further studies will be needed to decide if and how the threshold of 500 nM routinely being used as a threshold for peptide selection should be reconsidered.

The lack of experimental data is also among the causes of another potential problem. The datasets that are used to train these models are often very redundant: they contain many epitopes that are either identical or very similar. If not properly managed, redundancy can cause the tools to overfit: this means that their actual prediction accuracy on new data will be worse than the one reported in the publications. As a general suggestion, we encourage the users to check that the tools they are using take redundancy into account, for example by performing homology reduction procedures (87), rather than basing their choice on a purely numerical comparison of the accuracies reported in the papers.

A potentially very important but much less studied area is PTMs. Different PTMs exist such as phosphorylation, ubuiquitinylation, glycosylation, methylation, citrullination, to name a few. PTMs have been thought to be potential neoepitope candidates. This is based on the theory that peptides with aberrant PTMs have not been exposed to the immune system and thus potentially not subject to central tolerance. It has been shown that PTM self-antigens are capable of escaping central tolerance and being recognized by the immune system (88). Aberrant PTMs have been discovered in multiple cancers. Increased levels of glycans have for example been observed in cancers such as breast cancer (89, 90). However, identifying glycosylation sites as well as other PTM sites is not an easy task. In general mass spectrometry is often not capable of identifying less abundant proteins, due to its low sensitivity, thus capturing PTM information can be difficult due to the general low abundance.

Another lesser explored avenue are neoantigens derived from generally considered non-coding regions of the genome. Since they are less explored and studied, they are less utilized for analysis. Despite this, Laumont et al. (43) showed in their recent study that non-coding regions were possibly a considerable source of neoantigens.

There are still many events which are partially or completely disregarded by the current prediction models but can affect peptide binding and T cell recognition. Some examples include PTMs, local environment, self-similarity, clonality, and non-coding derived peptides. Moving forward, a tool which covers as many different neoepitope causing events as possible would be ideal. Another open question is whether some genomic aberrations are more effective than others for attacking the cancer cells. This begs the question of whether this is a generalized property or inherently specific for individual cancers, thus impairing the effectiveness of one-fits-all models.

Some of the tools presented in this review have been used in developing therapies that are being tested in ongoing clinical and pre-clinical trials. To mention a few, the development of neoantigen targeted personalized cancer treatments for cancers such as melanoma (91), glioblastoma (92) and non-small cell lung cancer (93) have been showing promising results. In particular, the use of tools that rely heavily on mhc binding prediction has propelled the discovery of candidates for test and use in targeted personalized immunotherapy in these studies. Even though these trials had encouraging results, they have also met some limitations in regards to the efficiency of the targeted immunotherapy, indicating that we are still in the early stages of development for neoepitope prediction tools. We envision that a growing amount of evidence on neoepitopes and on the ability of different tools to predict them will have a major impact on the development of better epitope and neoepitope prediction tools, and in turn help guide future immunotherapies.

## Author Contributions

A-LS-J and PM conceived and wrote the paper. MV created the figures together with A-LS-J and corrected and commented the paper. AB and SH corrected and commented the paper. All authors contributed to the article and approved the submitted version.

## Funding

A-LS-J is funded by the 2018 SDC grants.

## Conflict of Interest

The authors declare that the research was conducted in the absence of any commercial or financial relationships that could be construed as a potential conflict of interest.

## Publisher’s Note

All claims expressed in this article are solely those of the authors and do not necessarily represent those of their affiliated organizations, or those of the publisher, the editors and the reviewers. Any product that may be evaluated in this article, or claim that may be made by its manufacturer, is not guaranteed or endorsed by the publisher.

## References

[1] Burnet FM. Immunological Aspects of Neoplasia. In: Schwartz RS, editor. Prog Tumor Res. Basel, Karger (1970). vol 13, pp. 1-27. doi: 10.1159/000386035 4921480

[2] Thomas L. On Immunosurveillance in Human Cancer. Yale J Biol Med (1982) 55:329–33. doi: 10.18632/oncotarget.2998 PMC2596448 6758376

[3] Galluzzi L Vacchelli E Bravo-San Pedro JM Buqué A Senovilla L Baracco EE. Classification of Current Anticancer Immunotherapies. Oncotarget (2014) 5:12472–508. doi: 10.18632/oncotarget.2998 PMC4350348 25537519

[4] Humphries C. Adoptive Cell Therapy: Honing That Killer Instinct. Nature (2013) 504. doi: 10.1038/504S13a 24352359

[5] Nagasawa DT Fong C Yew A Spasic M Garcia HM Kruse CA. Passive Immunotherapeutic Strategies for the Treatment of Malignant Gliomas. Neurosurgery Clinics of North America (2012) 23(3):481-95. doi: 10.1016/j.nec.2012.04.008 PMC3994467 22748660

[6] Satoh Y Esche C Gambotto A Shurin GV Yurkovetsky ZR Robbins PD. Local Administration of IL-12-Transfected Dendritic Cells Induces Antitumor Immune Responses to Colon Adenocarcinoma in the Liver in Mice. J Exp Ther Oncol (2002) 2:337–49. doi: 10.1046/j.1359-4117.2002.01050.x 12440225

[7] Rice J Ottensmeier CH Stevenson FK. DNA Vaccines: Precision Tools for Activating Effective Immunity Against Cancer. Nat Rev Cancer (2008) 108-20. doi: 10.1038/nrc2326 18219306

[8] Singh-Jasuja H Emmerich NP Rammensee HG. The Tübingen Approach: Identification, Selection, and Validation of Tumor-Associated Hla Peptides for Cancer Therapy. Cancer Immunol Immunother (2004) 53:187–95. doi: 10.1007/s00262-003-0480-x PMC11032959 14758508

[9] Mommen GP Frese CK Meiring HD Gaans-van Den Brink J De Jong AP Van Els CA. Expanding the Detectable HLA Peptide Repertoire Using Electron-Transfer/Higher-Energy Collision Dissociation (EThcD). Proc Natl Acad Sci USA (2014) 111:4507–12. doi: 10.1073/pnas.1321458111 PMC3970485 24616531

[10] Falk K Rotzschke O Stevanovic S Jung G Rammenseet HG. Allele-Specific Motifs Revealed by Sequencing of Self-Peptides Eluted From MHC Molecules. Tech Rep (1991) 351:290–6. doi: 10.1038/351290a0 1709722

[11] Peters B Nielsen M. Sette A. T Cell Epitope Predictions. Annu Rev Immunol (2020) 38:123–45. doi: 10.1146/annurev-immunol-082119-124838 PMC10878398 32045313

[12] Tung CW Ho SY. POPI: Predicting Immunogenicity of MHC Class I Binding Peptides by Mining Informative Physicochemical Properties. Bioinformatics (2007) 23:942–9. doi: 10.1093/bioinformatics/btm061 17384427

[13] Tung CW Ziehm M Kämper A Kohlbacher O Ho SY. POPISK: T-Cell Reactivity Prediction Using Support Vector Machines and String Kernels. BMC Bioinf (2011) 12:446. doi: 10.1186/1471-2105-12-446 PMC3228774 22085524

[14] Calis JJ Maybeno M Greenbaum JA Weiskopf D De Silva AD Sette A. Properties of MHC Class I Presented Peptides That Enhance Immunogenicity. PloS Comput Biol (2013) 9:1003266. doi: 10.1371/journal.pcbi.1003266 PMC3808449 24204222

[15] Saethang T Hirose O Kimkong I Tran VA Dang XT Nguyen LAT. PAAQD: Predicting Immunogenicity of MHC Class I Binding Peptides Using Amino Acid Pairwise Contact Potentials and Quantum Topological Molecular Similarity Descriptors. J Immunol Methods (2013) 387:293–302. doi: 10.1016/j.jim.2012.09.016 23058674

[16] Jørgensen KW Rasmussen M Buus S Nielsen M. NetMHCstab - Predicting Stability of Peptide-MHC-I Complexes; Impacts for Cytotoxic T Lymphocyte Epitope Discovery. Immunology (2014) 141:18–26. doi: 10.1111/imm.12160 23927693 PMC3893846

[17] Rasmussen M Fenoy E Harndahl M Kristensen AB Nielsen IK Nielsen M. Pan-Specific Prediction of Peptide–MHC Class I Complex Stability, a Correlate of T Cell Immunogenicity. J Immunol (2016) 197:1517–24. doi: 10.4049/jimmunol.1600582 PMC4976001 27402703

[18] Schmidt J Guillaume P Dojcinovic D Karbach J Coukos G Luescher I. In Silico and Cell-Based Analyses Reveal Strong Divergence Between Prediction and Observation of T-Cell-Recognized Tumor Antigen T-Cell Epitopes. J Biol Chem (2017) 292:11840–9. doi: 10.1074/jbc.M117.789511 PMC5512077 28536262

[19] Trolle T Nielsen M. NetTepi: An Integrated Method for the Prediction of T Cell Epitopes. Immunogenetics (2014) 66:449–56. doi: 10.1007/s00251-014-0779-0 24863339

[20] Karosiene E Lundegaard C Lund O Nielsen M. NetMHCcons: A Consensus Method for the Major Histocompatibility Complex Class I Predictions. Immunogenetics (2012) 64:177–86. doi: 10.1007/s00251-011-0579-8 22009319

[21] Jurtz VI Jessen LE Bentzen AK Jespersen MC Mahajan S Vita R. NetTCR: Sequence-Based Prediction of TCR Binding to Peptide-MHC Complexes Using Convolutional Neural Networks. bioRxiv (2018). doi: 10.1101/433706

[22] Rammensee HG Bachmann J Emmerich NPN Bachor OA Stevanović S. SYFPEITHI: Database for MHC Ligands and Peptide Motifs. Immunogenetics (1999) 50:213-9. doi: 10.1007/s002510050595 10602881

[23] Fleri W Paul S Dhanda SK Mahajan S Xu X Peters B. The Immune Epitope Database and Analysis Resource in Epitope Discovery and Synthetic Vaccine Design. Front Immunol (2017). doi: 10.3389/fimmu.2017.00278 PMC5348633 28352270

[24] Bagaev DV Vroomans RM Samir J Stervbo U Rius C Dolton G. VDJdb in 2019: Database Extension, New Analysis Infrastructure and a T-Cell Receptor Motif Compendium. Nucleic Acids Res (2020) 48:D1057–62. doi: 10.1093/nar/gkz874 PMC6943061 31588507

[25] Tickotsky N Sagiv T Prilusky J Shifrut E Friedman N. McPAS-TCR: A Manually Curated Catalogue of Pathology-Associated T Cell Receptor Sequences. Bioinformatics (2017) 33:2924–9. doi: 10.1093/bioinformatics/btx286 28481982

[26] Borrman T Cimons J Cosiano M Purcaro M Pierce BG Baker BM. ATLAS: A Database Linking Binding Affinities With Structures for Wild-Type and Mutant TCR-pMHC Complexes. Proteins: Struct Funct Bioinf (2017) 85:908–16. doi: 10.1002/prot.25260 PMC5860664 28160322

[27] Leem J De Oliveira SH Krawczyk K Deane CM. STCRDab: The Structural T-Cell Receptor Database. Nucleic Acids Res (2018) 46:D406–12. doi: 10.1093/nar/gkx971 PMC5753249 29087479

[28] Rudolph MG Wilson IA. The Specificity of TCR/pMHC Interaction. Current Opinion Immun (2002) 14(1):52-65. doi: 10.1016/S0952-7915(01)00298-9 11790533

[29] Bentzen AK Such L Jensen KK Marquard AM Jessen LE Miller NJ. T Cell Receptor Fingerprinting Enables in-Depth Characterization of the Interactions Governing Recognition of Peptide–MHC Complexes. Nat Biotechnol (2018) 36:1191–6. doi: 10.1038/nbt.4303 PMC9452375 30451992

[30] Knapp B. Deane CM. T-Cell Receptor Binding Affects the Dynamics of the Peptide/MHC-I Complex. J Chem Inf Model (2016) 56:46–53. doi: 10.1021/acs.jcim.5b00511 26633740

[31] Qi Q Liu Y Cheng Y Glanville J Zhang D Lee JY. Diversity and Clonal Selection in the Human T-Cell Repertoire. Proc Natl Acad Sci USA (2014) 111:13139–44. doi: 10.1073/pnas.1409155111 PMC4246948 25157137

[32] Jenkins MK Chu HH McLachlan JB Moon JJ. On the Composition of the Preimmune Repertoire of T Cells Specific for Peptide–Major Histocompatibility Complex Ligands. Annu Rev Immunol (2010) 28:275–94. doi: 10.1146/annurev-immunol-030409-101253 20307209

[33] Jensen KK Rantos V Jappe EC Olsen TH Jespersen MC Jurtz V. TCRpMHCmodels: Structural Modelling of TCR-pMHC Class I Complexes. Sci Rep (2019) 9. doi: 10.1038/s41598-019-50932-4 PMC6787230 31601838

[34] Gielis S Moris P Bittremieux W De Neuter N Ogunjimi B Laukens K. Detection of Enriched T Cell Epitope Specificity in Full T Cell Receptor Sequence Repertoires. Front Immunol (2019) 10:2820. doi: 10.3389/fimmu.2019.02820 31849987 PMC6896208

[35] Li S Wilamowski J Teraguchi S van Eerden FJ Rozewicki J Davila A. Structural Modeling of Lymphocyte Receptors and Their Antigens. Methods Mol Biol (Humana Press Inc) (2019) 2048:207–29. doi: 10.1007/978-1-4939-9728-2_17 31396940

[36] Lanzarotti E Marcatili P Nielsen M. Identification of the Cognate Peptide-MHC Target of T Cell Receptors Using Molecular Modeling and Force Field Scoring. Mol Immunol (2018) 94:91–7. doi: 10.1016/j.molimm.2017.12.019 PMC5800965 29288899

[37] Lanzarotti E Marcatili P Nielsen M. T-Cell Receptor Cognate Target Prediction Based on Paired *α* and *β* Chain Sequence and Structural CDR Loop Similarities. Front Immunol (2019) 10:2080. doi: 10.3389/fimmu.2019.02080 31555288 PMC6724566

[38] Simons KT Bonneau R Ruczinski I Baker D. Ab Initio Protein Structure Prediction of CASP III Targets Using ROSETTA. Proteins (1999) Suppl 3. doi: 10.1002/(SICI)1097-0134(1999)37:3+<171::AID-PROT21>3.3.CO;2-Q 10526365

[39] Schymkowitz J Borg J Stricher F Nys R Rousseau F Serrano L. The FoldX Web Server: An Online Force Field. Nucleic Acids Res (2005) 33:W382–8. doi: 10.1093/nar/gki387 PMC1160148 15980494

[40] Aranha MP Jewel YSM Beckman RA Weiner LM Mitchell JC Parks JM. Combining Three-Dimensional Modeling With Artificial Intelligence to Increase Specificity and Precision in Peptide–MHC Binding Predictions. J Immunol (2020) 205:1962–77. doi: 10.4049/jimmunol.1900918 PMC7511449 32878910

[41] Alexandrov LB Nik-Zainal S Wedge DC Aparicio SA Behjati S Biankin AV. Signatures of Mutational Processes in Human Cancer. Nature (2013) 500:415–21. doi: 10.1038/nature12477 PMC3776390 23945592

[42] Malaker SA Penny SA Steadman LG Myers PT Loke JC Raghavan M. Identification of Glycopeptides as Posttranslationally Modified Neoantigens in Leukemia. Cancer Immun Res (2017) 5(5):376–84. doi: 10.1158/2326-6066.CIR-16-0280 PMC5508727 28314751

[43] Laumont CM Vincent K Hesnard L Audemard R Bonneil R Laverdure JP. Noncoding Regions Are the Main Source of Targetable Tumor-Specific Antigens. Sci Trans Med (2018) 10. doi: 10.1126/scitranslmed.aau5516 30518613

[44] Solleder M Guillaume P Racle J Michaux J Pak HS Müller M. Mass Spectrometry Based Immunopeptidomics Leads to Robust Predictions of Phosphorylated Hla Class I Ligands. Mol Cell Proteomics (2020) 19:390–404. doi: 10.1074/mcp.TIR119.001641 31848261 PMC7000122

[45] Wickström SL Lövgren T Volkmar M Reinhold B Duke-Cohan JS Hartmann L. Cancer Neoepitopes for Immunotherapy: Discordance Between Tumor-Infiltrating T Cell Reactivity and Tumor MHC Peptidome Display. Front Immunol (2019) 10:2766. doi: 10.3389/fimmu.2019.02766 31921104 PMC6918724

[46] Smart AC Margolis CA Pimentel H He MX Miao D Adeegbe D. Intron Retention Is a Source of Neoepitopes in Cancer. Nat Biotechnol (2018) 36:1056–63. doi: 10.1038/nbt.4239 PMC6226333 30114007

[47] Grade M Difilippantonio MJ Camps J. Patterns of Chromosomal Aberrations in Solid Tumors. Chromosomal Instability Cancer Cells (Springer Int Publ) (2015) 200:115–42. doi: 10.1007/978-3-319-20291-4_6 PMC4729311 26376875

[48] Wei Z Zhou C Zhang Z Guan M Zhang C Liu Z. The Landscape of Tumor Fusion Neoantigens: A Pan-Cancer Analysis. iScience (2019) 21:249–60. doi: 10.1016/j.isci.2019.10.028 PMC6838548 31677477

[49] Duan F Duitama J Al Seesi S Ayres CM Corcelli SA Pawashe AP. Genomic and Bioinformatic Profiling of Mutational Neoepitopes Reveals New Rules to Predict Anticancer Immunogenicity. J Exp Med (2014) 211:2231–48. doi: 10.1084/jem.20141308 PMC4203949 25245761

[50] Tappeiner E Finotello F Charoentong P Mayer C Rieder D Trajanoski Z. TIminer: NGS Data Mining Pipeline for Cancer Immunology and Immunotherapy. Bioinformatics (2017) 33:3140–1. doi: 10.1093/bioinformatics/btx377 PMC5870678 28633385

[51] Kim S Kim HS Kim E Lee MG Shin EC Paik S. Neopepsee: Accurate Genome-Level Prediction of Neoantigens by Harnessing Sequence and Amino Acid Immunogenicity Information. Ann Oncol (2018) 29:1030–6. doi: 10.1093/annonc/mdy022 29360924

[52] Shi Y Guo Z Su X Meng L Zhang M Sun J. DeepAntigen: A Novel Method for Neoantigen Prioritization *via* 3D Genome and Deep Sparse Learning. Bioinformatics (2020) 36(19):4894–901. doi: 10.1093/bioinformatics/btaa596 32592462

[53] Hundal J Carreno BM Petti AA Linette GP Griffith OL Mardis ER. pVAC-Seq: A Genome-Guided *In Silico* Approach to Identifying Tumor Neoantigens. Genome Med (2016) 8:11. doi: 10.1186/s13073-016-0264-5 26825632 PMC4733280

[54] Bjerregaard AM Nielsen M Hadrup SR Szallasi Z Eklund AC. MuPeXI: Prediction of Neo-Epitopes From Tumor Sequencing Data. Cancer Immunol Immunother (2017) 66:1123–30. doi: 10.1007/s00262-017-2001-3 PMC11028452 28429069

[55] Mondet S Aksoy BA Rozenberg L Hodes I Hammerbacher J. Bioinformatics Workflow Management With the Wobidisco Ecosystem. bioRxiv (2017). doi: 10.1101/213884

[56] Kodysh J Rubinsteyn A. OpenVax: An Open-Source Computational Pipeline for Cancer Neoantigen Prediction. Methods Mol Biol (Humana Press Inc) (2020) 2120:147–60. doi: 10.1007/978-1-0716-0327-7_10 32124317

[57] Wood MA Nguyen A Struck AJ Ellrott K Nellore A Thompson RF. Neoepiscope Improves Neoepitope Prediction With Multivariant Phasing. Bioinformatics (2020) 36:713–20. doi: 10.1093/bioinformatics/btz653 31424527

[58] Bais P Namburi S Gatti DM Zhang X Chuang JH. CloudNeo: A Cloud Pipeline for Identifying Patient-Specific Tumor Neoantigens. Bioinformatics (2017) 33:3110–2. doi: 10.1093/bioinformatics/btx375 PMC5870764 28605406

[59] Zhou C Wei Z Zhang Z Zhang B Zhu C Chen K. PTuneos: Prioritizing Tumor Neoantigens From Next-Generation Sequencing Data. Genome Med (2019) 11:67. doi: 10.1186/s13073-019-0679-x 31666118 PMC6822339

[60] Richman LP Vonderheide RH Rech AJ. Neoantigen Dissimilarity to the Self-Proteome Predicts Immunogenicity and Response to Immune Checkpoint Blockade. Cell Syst (2019) 9:375–82. doi: 10.1016/j.cels.2019.08.009 PMC6813910 31606370

[61] Schenck RO Lakatos E Gatenbee C Graham TA Anderson AR. NeoPredPipe: High-Throughput Neoantigen Prediction and Recognition Potential Pipeline. BMC Bioinf (2019) 20:264. doi: 10.1186/s12859-019-2876-4 PMC6532147 31117948

[62] Zhou Z Lyu X Wu J Yang X Wu S Zhou J. TSNAD: An Integrated Software for Cancer Somatic Mutation and Tumour-Specific Neoantigen Detection. R Soc Open Sci (2017) 4(4):170050. doi: 10.1098/rsos.170050 28484631 PMC5414268

[63] Wang TY Wang L Alam SK Hoeppner LH Yang R. ScanNeo: Identifying Indel-Derived Neoantigens Using RNA-Seq Data. Bioinformatics (2019) 35:4159–61. doi: 10.1093/bioinformatics/btz193 30887025

[64] Fotakis G Rieder D Haider M Trajanoski Z Finotello F. NeoFuse: Predicting Fusion Neoantigens From RNA Sequencing Data. Bioinformatics (2020) 36:2260–1. doi: 10.1093/bioinformatics/btz879 PMC7141848 31755900

[65] Zhang J Mardis ER Maher CA. INTEGRATE-Neo: A Pipeline for Personalized Gene Fusion Neoantigen Discovery. Bioinformatics (2017) 33:555–7. doi: 10.1093/bioinformatics/btw674 PMC5408800 27797777

[66] Bulik-Sullivan B Busby J Palmer CD Davis MJ Murphy T Clark A. Deep Learning Using Tumor HLA Peptide Mass Spectrometry Datasets Improves Neoantigen Identification. Nat Biotechnol (2019) 37:55–71. doi: 10.1038/nbt.4313 30556813

[67] Wu J Wang W Zhang J Zhou B Zhao W Su Z. DeepHLApan: A Deep Learning Approach for Neoantigen Prediction Considering Both HLA-Peptide Binding and Immunogenicity. Front Immunol (2019) 10:2559. doi: 10.3389/fimmu.2019.02559 31736974 PMC6838785

[68] Nielsen M Lundegaard C Worning P Lauemøller SL Lamberth K Buus S. Reliable Prediction of T-Cell Epitopes Using Neural Networks With Novel Sequence Representations. Protein Sci (2003) 12:1007–17. doi: 10.1110/ps.0239403 PMC2323871 12717023

[69] Nielsen M Lundegaard C Blicher T Lamberth K Harndahl M Justesen S. NetMHCpan, a Method for Quantitative Predictions of Peptide Binding to Any HLA-A and -B Locus Protein of Known Sequence. PloS One (2007) 2:e796. doi: 10.1371/journal.pone.0000796 17726526 PMC1949492

[70] Nicolae M Mangul S Mǎndoiu II Zelikovsky A. Estimation of Alternative Splicing Isoform Frequencies From RNA-Seq Data. Algorithms Mol Biol (2011) 6:9. doi: 10.1186/1748-7188-6-9 21504602 PMC3107792

[71] Pan Q Shai O Lee LJ Frey BJ Blencowe BJ. Deep Surveying of Alternative Splicing Complexity in the Human Transcriptome by High-Throughput Sequencing. Nat Genet (2008) 40:1413–5. doi: 10.1038/ng.259 18978789

[72] Pickrell JK Marioni JC Pai AA Degner JF Engelhardt BE Nkadori E. Understanding Mechanisms Underlying Human Gene Expression Variation With RNA Sequencing. Nature (2010) 464:768–72. doi: 10.1038/nature08872 PMC3089435 20220758

[73] O’Donnell TJ Rubinsteyn A Bonsack M Riemer AB Laserson U Hammerbacher J. MHCflurry: Open-Source Class I MHC Binding Affinity Prediction. Cell Syst (2018) 7:129–32. doi: 10.1016/j.cels.2018.05.014 29960884

[74] Abelin JG Keskin DB Sarkizova S Hartigan CR Zhang W Sidney J. Mass Spectrometry Profiling of HLA-Associated Peptidomes in Mono-Allelic Cells Enables More Accurate Epitope Prediction. Immunity (2017) 46:315–26. doi: 10.1016/j.immuni.2017.02.007 PMC5405381 28228285

[75] Warren RL Choe G Freeman DJ Castellarin M Munro S Moore R. Derivation of HLA Types From Shotgun Sequence Datasets. Genome Med (2012) 4:95. doi: 10.1186/gm396 23228053 PMC3580435

[76] Zhang J White NM Schmidt HK Fulton RS Tomlinson C Warren WC. INTEGRATE: Gene Fusion Discovery Using Whole Genome and Transcriptome Data. Genome Res (2016) 26:108–18. doi: 10.1101/gr.186114.114 PMC4691743 26556708

[77] van Berkum NL Lieberman-Aiden E Williams L Imakaev M Gnirke A Mirny LA. Hi-C: A Method to Study the Three-Dimensional Architecture of Genomes. J Vis Exp (2010) (39):e1869. doi: 10.3791/1869 PMC3149993 20461051

[78] Yewdell JW Bennink JR. Immunodominance in Major Histocompatibility Complex Class I-Restricted T Lymphocyte Responses. Annu Rev Immunol (1999) 17:51–88. doi: 10.1146/annurev.immunol.17.1.51 10358753

[79] Sette A Vitiello A Reherman B Fowler P Nayersina R Kast WM. The Relationship Between Class I Binding Affinity and Immunogenicity of Potential Cytotoxic T Cell Epitopes. J Immunol (1994) 153:5586–92. 7527444

[80] Croft NP Smith SA Pickering J Sidney J Peters B Faridi P. Most Viral Peptides Displayed by Class I MHC on Infected Cells Are Immunogenic. Proc Natl Acad Sci USA (2019) 116:3112–7. doi: 10.1073/pnas.1815239116 PMC6386720 30718433

[81] Zhong W Reche PA Lai CC Reinhold B Reinherz EL. Genome-Wide Characterization of a Viral Cytotoxic T Lymphocyte Epitope Repertoire. J Biol Chem (2003) 278:45135–44. doi: 10.1074/jbc.M307417200 12960169

[82] Dönnes P Kohlbacher O. Integrated Modeling of the Major Events in the MHC Class I Antigen Processing Pathway. Protein Sci (2005) 14:2132–40. doi: 10.1110/ps.051352405 PMC2279325 15987883

[83] Bjerregaard AM Nielsen M Jurtz V Barra CM Hadrup SR Szallasi Z. An Analysis of Natural T Cell Responses to Predicted Tumor Neoepitopes. Front Immunol (2017) 8:1566. doi: 10.3389/fimmu.2017.01566 29187854 PMC5694748

[84] Koşaloğlu-Yalçın Z Lanka M Frentzen A Logandha Ramamoorthy Premlal A Sidney J Vaughan K. Predicting T Cell Recognition of MHC Class I Restricted Neoepitopes. OncoImmunology (2018) 7:e1492508. doi: 10.1080/2162402X.2018.1492508 30377561 PMC6204999

[85] Fritsch EF Rajasagi M Ott PA Brusic V Hacohen N Wu CJ. HLA-Binding Properties of Tumor Neoepitopes in Humans. Cancer Immun Res (2014) 2(6). doi: 10.1158/2326-6066.CIR-13-0227 PMC4049249 24894089

[86] Ghorani E Rosenthal R McGranahan N Reading JL Lynch M Peggs KS. Differential Binding Affinity of Mutated Peptides for MHC Class I Is a Predictor of Survival in Advanced Lung Cancer and Melanoma. Ann Oncol (2018) 29:271–9. doi: 10.1093/annonc/mdx687 PMC5834109 29361136

[87] Lund O Frimand K Gorodkin J Bohr H Bohr J Hansen J. Protein Distance Constraints Predicted by Neural Networks and Probability Density Functions. Protein Eng (1997) 10:1242–8. doi: 10.1093/protein/10.11.1241 9514112

[88] Raposo B Merky P Lundqvist C Yamada H Urbonaviciute V Niaudet C. T Cells Specific for Post-Translational Modifications Escape Intrathymic Tolerance Induction. Nat Commun (2018) 9(1):353. doi: 10.1038/s41467-017-02763-y 29367624 PMC5783942

[89] de Leoz MLA Young LJT An HJ Kronewitter SR Kim J Miyamoto S. High-Mannose Glycans Are Elevated During Breast Cancer Progression. Mol Cell Proteomics (2011) 10.(1):M110.002717 doi: 10.1074/mcp.m110.002717 PMC3013453 21097542

[90] Tesařová P Kalousová M Trnková B Soukupová J Argalášová S Mestek O. Carbonyl and Oxidative Stress in Patients With Breast Cancer-Is There a Relation to the Stage of the Disease? Tech Rep (2007) 54:219–24. 17447853

[91] Ott PA Hu Z Keskin DB Shukla SA Sun J Bozym DJ. An Immunogenic Personal Neoantigen Vaccine for Patients With Melanoma. Nature (2017) 547:217–21. doi: 10.1038/nature22991 PMC5577644 28678778

[92] Keskin DB Anandappa AJ Sun J Tirosh I Mathewson ND Li S. Neoantigen Vaccine Generates Intratumoral T Cell Responses in Phase Ib Glioblastoma Trial. Nature (2019) 565:234–9. doi: 10.1038/s41586-018-0792-9 PMC6546179 30568305

[93] Zhang W Yin Q Huang H Lu J Qin H Chen S. Personal Neoantigens From Patients With NSCLC Induce Efficient Antitumor Responses. Front Oncol (2021) 11:628456. doi: 10.3389/fonc.2021.628456 33928024 PMC8076796

